# Discussion by the British Hospitals Association

**Published:** 1921-02-19

**Authors:** 


### NURSES' REMUNERATION AND THE COLLEGE SCALE.

### Discussion by The British Hospitals Association.

The revised scale of salaries recommended by the College of Nursing was the subject of a lively discussion by the Midlands Regional Committee of the British Hospitals Association at a meeting held on February 10 at the General Hospital, Birmingham. Mr. C. H. Saunders (Chairman of the General Hospital, Birmingham) occupied the chair, and the meeting was attended by representatives of about twenty-three hospitals throughout the Midlands.

Much sympathy was expressed with the efforts of the College of Nursing to bring about a standardisation of nurses' salaries, but the general feeling was that the recent proposals exceeded the limits of reasonable demands, and that an inopportune time had been chosen for launching the scheme. Mr. Tom B. Adams (Chairman of the Wolverhampton General Hospital) said that as far as hospitals were concerned there was never any opportune time for such proposals! He thought, however, that nurses at the present time are inadequately remunerated for the services they render; but the College of Nursing had gone a good deal too far and were asking too much—more, in fact, than hospitals could possibly pay, especially when faced by the necessity of increasing their nursing staffs through shorter hours on duty. The proposed scale must have been considered and brought forth at a time when the country was at the high-water mark of costs, and is more than a nurse is entitled to receive or a hospital to pay. The time of the abnormally high wages in other walks of life has now passed.

It was agreed that it is imperative to pay nurses an adequate salary so as to attract the right type of woman, and it is obviously unthinkable that the poverty of the hospitals throughout the country should be thrust upon the nursing staff. It was pointed out that under the new scale a ward sister would receive £85 per annum, or £1 12s. 8d. per week, and for a sister in charge of thirty beds this would work out at less than 2d. per bed per day. No one could say that this salary is an extravagant one.

The Chairman said that he felt that the present rates for probationers were quite adequate, and deprecated the high rates of pay in some of the Poor-law hospitals for probationers whilst training. He expressed the opinion that the salary of matrons might well be left to the discretion of their individual Boards.

On behalf of the nurses, however, it was pointed out by several representatives that the lack of a uniform scale of salaries is one of the causes of the present dearth of the right type of nurse throughout the country, and a possible remedy for this would be the standardisation of salaries in voluntary hospitals as well as Poor-law infirmaries, thus enabling girls, when choosing a career, to review with some degree of accuracy the prospects of nursing as a profession.

The following resolution was finally put to the meeting and carried:—

"That the Midlands Regional Committee of the British Hospitals Association, whilst fully desirous of giving the nurses adequate remuneration for their services, consider that the proposed increase in the scale of salaries recommended by the College of Nursing in their circular dated December 1920 is undesirable in the present financial position of the hospitals."

In regard to the new telephone charges, it was resolved that a strong protest be made against the proposed increases, and that the British Hospitals Association be asked to endeavour to obtain special rates for all hospitals. It was stated that special rates had been granted to hospitals in the past, and there seems no apparent reason why the concession cannot again be made.

It was decided to nominate three representatives and ask permission for them to be allowed to give evidence before the Committee when the position of the provincial hospitals is considered.

Protest against Dangerous Drugs Regulations.

The proposed regulations under the Dangerous Drugs Act were considered, and an emphatic protest made against some of their provisions. The following resolution was carried unanimously:—

"That the Midlands Regional Committee of the British Hospitals Association protest against the proposed regulations under the Dangerous Drugs Act, and demand that they be withdrawn, and that a copy of this resolution be sent to the members of Parliament for the districts covered by the Committee."

Mr. Thomas Ratcliff (Chairman of the Finance Committee at the General Hospital, Birmingham) was elected a representative to serve on the Council of the British Hospitals Association in London.

